# Does B Cell Receptor Signaling in Chronic Lymphocytic Leukaemia Cells Differ from That in Other B Cell Types?

**DOI:** 10.1155/2014/208928

**Published:** 2014-07-02

**Authors:** Joseph R. Slupsky

**Affiliations:** Department of Molecular and Clinical Cancer Medicine, University of Liverpool, 6th Floor, Duncan Building, Daulby Street, Liverpool L69 3GA, UK

## Abstract

Chronic lymphocytic leukaemia (CLL) is an incurable malignancy of mature B cells. CLL is important clinically in Western countries because of its commonality and because of the significant morbidity and mortality associated with the progressive form of this incurable disease. The B cell receptor (BCR) expressed on the malignant cells in CLL contributes to disease pathogenesis by providing signals for survival and proliferation, and the signal transduction pathway initiated by engagement of this receptor is now the target of several therapeutic strategies. The purpose of this review is to outline current understanding of the BCR signal cascade in normal B cells and then question whether this understanding applies to CLL cells. In particular, this review studies the phenomenon of anergy in CLL cells, and whether certain adaptations allow the cells to overcome anergy and allow full BCR signaling to take place. Finally, this review analyzes how BCR signals can be therapeutically targeted for the treatment of CLL.

## 1. Introduction

Chronic lymphocytic leukaemia (CLL) is a common malignancy of mature B cells, accounting for 34% of all haematological cancers within the UK [1]. It represents a clinically important burden because there is significant morbidity and mortality associated with this disease, and currently it is incurable. Typically a patient with progressive disease will undergo several rounds of treatment and relapse before succumbing to the suppression of immune function and haemopoiesis that result from expansion of the malignant cells in hemic tissues. At the present time existing treatments focus on the prevention of disease progression and relief of symptoms [2]. However, new treatments specifically targeting the signaling pathway initiated following B cell antigen receptor (BCR) engagement are showing promise [3–5] and may lead to successful treatment of this disease. This review will assess current understanding of the BCR signaling pathway as it applies to CLL cells and discuss the potential for new therapies based on this understanding.

It is widely accepted that signals generated by engagement of the BCR play an important role in the pathogenesis of CLL [6]. For instance, it is known that the structures of BCR expressed on CLL cells from different patients can resemble each other to a high degree, indicating that antigens of a similar nature drive development of the disease [7, 8]. The structure of the antigen binding domain of BCR expressed on CLL cells is biased towards a select number of immunoglobulin heavy-chain (IGHV) gene segments that have been rearranged in a very restricted manner [8, 9]. In particular, the heavy-chain complementarity-determining region (HCDR3) is longer than average [9, 10], and this feature has recently been demonstrated to play a role in cell-autonomous antigen-independent BCR signaling in CLL cells [11]. Furthermore, usage of IGHV genes such as 3–21 and 1–69 is associated with poor disease prognosis, whereas usage of genes such as 4–34 and 2–30 is associated with indolent disease [12]. Further difference between patients with progressive versus indolent disease appears to reside in specificity of antigen binding by the BCR, and this is defined by the degree to which mutation of the genes coding for a IGHV gene has affected the germline sequence. Those CLL patients which have malignant cells bearing IGHV gene sequences with greater than 98% homology to the germline sequence are termed unmutated CLL (UM-CLL), and those with less are termed mutated CLL (M-CLL). This is important because the BCR on M-CLL cells shows restricted antigen specificity compared to that on UM-CLL cells. Practically, this means that the BCR on different CLL cell clones may use the same IGHV genes, but the clone with unmutated genes will be polyreactive whereas the clone with mutated genes will be more monoreactive [13]. Thus, the polyreactivity of the BCR on UM-CLL cells allows binding to a variety of self and foreign antigens [14], and constitutive signals generated by this engagement are thought to contribute to disease progression. Proof of* in vivo* BCR engagement is suggested by Krysov et al. [15], who have shown that the BCR expressed on CLL cells, particularly from UM-CLL patients, has features that are associated with continuous* in vivo* exposure to antigen. Others have demonstrated that such* in vivo* BCR stimulation is reflected in the pattern of gene expression observed in freshly isolated cells [16]. Taken together, these observations suggest that targeting continuous BCR signaling may show therapeutic benefit in CLL patients with progressive disease, and this notion is now turned into a therapeutic strategy. New drugs that inhibit kinases within the BCR signaling pathway, notably ibrutinib (an inhibitor of Bruton's tyrosine kinase (Btk)), fostamatinib (an inhibitor of Syk), and idelalisib (an inhibitor of phosphatidylinositol 3 kinase *δ* (PI3K*δ*)), all appear to show promise in clinical studies [3–5].

## 2. BCR Signaling in Normal B Cells

**Figure 1:**
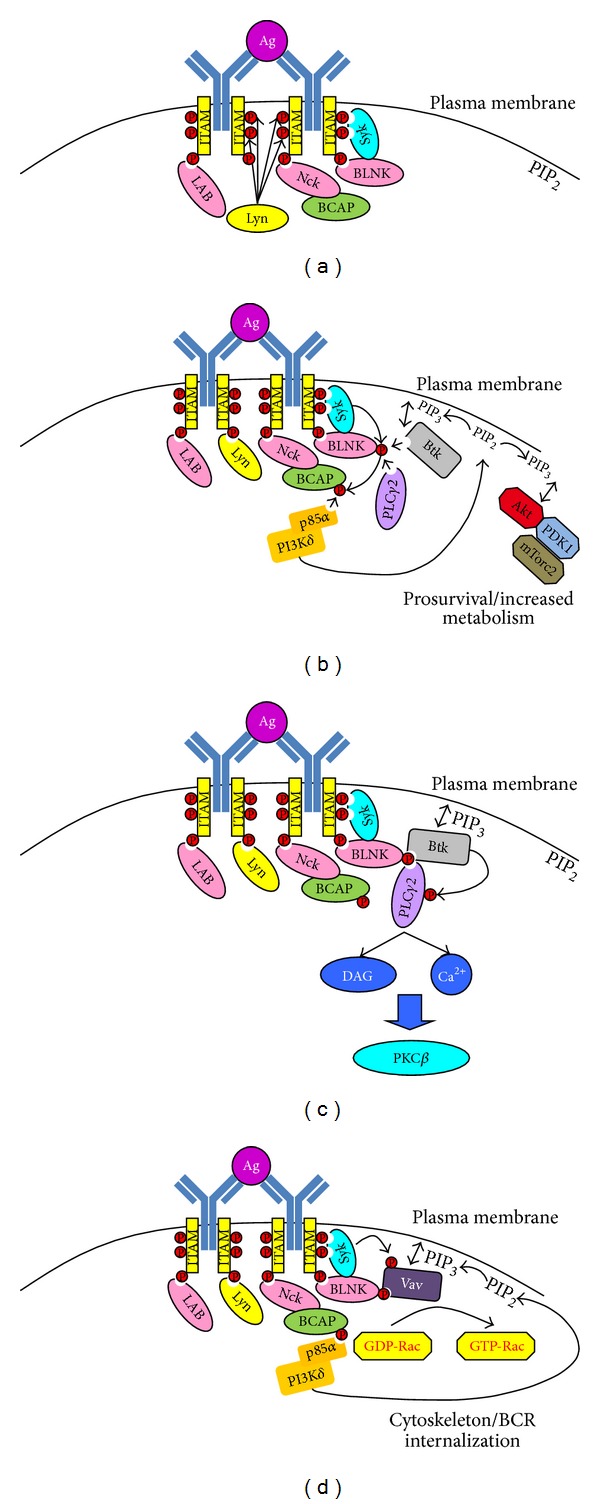
Induction of proximal BCR signaling. Illustration of the most proximal signals initiated during BCR engagement. (a) Antigen engagement of the BCR induces Lyn-mediated phosphorylation of CD79 on tyrosine residues within and outside the ITAM motif. This attracts adaptor molecules such as LAB, Nck, BCAP, and BLNK, as well as the tyrosine kinase Syk. (b) Syk binding to tyrosine phosphorylated CD79 induces its activation, and it phosphorylates BLNK, BCAP, and LAB. Phospho-BCAP attracts and activates PI3K*δ* and converts PIP_2_ to PIP_3_. The presence of PIP_3_ on the plasma membrane attracts PDK1 and Akt; PDK1 phosphorylates and activates Akt, and this is followed by a second phosphorylation event by mTorc2. PIP_3_ on the plasma membrane also attracts Btk, which then binds to phospho-BLNK and exposes a phosphorylation site for Syk leading to autophosphorylation and full activation of Btk [17]. Phospho-BLNK also acts as a scaffold for PLC*γ*2. (c) Active Btk phosphorylates and activates PLC*γ*2 and catalyzes the conversion of PIP_2_ to DAG and IP_3_ (which acts on ER to release intracellular Ca^2+^). DAG and Ca^2+^ then act to activate PKC*β*. (d) Phospho-BLNK and phospho-LAB serve as scaffolds for Vav, which is also attracted to the plasma membrane by the presence of PIP_3_. Syk is then able to phosphorylate and activate Vav, which then acts as a guanine exchange factor to convert GDP-Rac1/2 to GTP-Rac1/2. This results in cytoskeletal changes and induction of BCR internalization.

Our understanding of BCR signaling in CLL cells is based on a model that has been developed over many years.  Figure 1 illustrates the signal transduction pathway that is activated by BCR stimulation [18]. Thus, upon BCR engagement the two tyrosine residues within the immune-receptor tyrosine-based activation motifs (ITAMs) of CD79a become phosphorylated by Src-family kinases (SFKs) [19, 20], specifically by Lyn, Fyn, and Blk, the three most abundant SFKs in B cells (Figure 1(a)) [21]. Phosphorylation of these two tyrosine residues in CD79 creates a binding site for the tandem SRC-homology 2 (SH2) motifs within Syk [22], and this binding induces activation and phosphorylation of Syk [23]. Further propagation of the BCR signal is achieved by recruiting the adaptor proteins B cell linker (BLNK, SLP-65), linker of activation in B cells (LAB), and Nck to phosphotyrosine residues outside the ITAMs of CD79a and b [24–26]. Once bound to CD79, BLNK becomes a substrate for Syk [24], and this allows the recruitment of additional proteins such as Btk, phospholipase C*γ*2 (PLC*γ*2), Grb2, and Vav to the developing signalosome [27]. Nck recruits another adaptor protein, B cell adaptor for PI3K (BCAP), which becomes phosphorylated by Syk and acts to recruit and activate PI3K*δ* by binding its associated p85*α* regulatory subunit (Figure 1(b)) [28, 29]. Active PI3K*δ* then provides phosphatidylinositol 3,4,5-trisphosphate (PIP_3_) to facilitate the recruitment of PDK1, Akt, Btk, PLC*γ*2, and Vav to the plasma membrane via their pleckstrin homology (PH) domains. The close juxtaposition of PDK1 with Akt stimulates threonine 308 phosphorylation and activation of Akt kinase function that is then stabilized by phosphorylation of serine 473 by mTorc2 [30]. The close juxtaposition of Syk, Btk, and PLC*γ*2 at the B cell plasma membrane allows Syk and Btk to phosphorylate PLC*γ*2 and induce its enzymatic activation (Figure 1(c)) [31].

**Figure 2:**
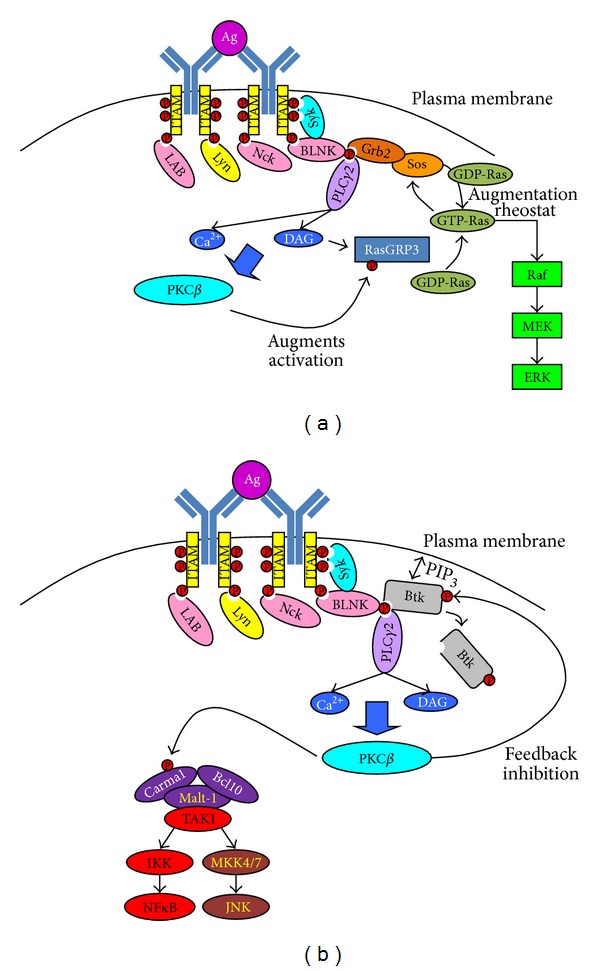
Signal integration following BCR engagement. Schematic of distal signaling events following BCR engagement. (a) Phospho-BLNK serves as a scaffold for Grb2, and this acts to prime activation of Sos. Active PKC*β* phosphorylates RasGRP3 to facilitate its activation by DAG. The guanine exchange factor function of RasGRP3 converts GDP-Ras to GTP-Ras, which then acts to augment the guanine exchange factor function of Sos. Together RasGRP3 and Sos produce sufficient GTP-Ras to power activation of the MAPK cascade illustrated here by Raf, MEK, and ERK. (b) Active PKC*β* acts to phosphorylate CARMA1 to induce assembly of the CARMA1-Bcl10-MALT1 complex. This allows activation of TAK1 which facilitates activation of the NF*κ*B pathway by phosphorylating IKK and of the JNK pathway by phosphorylating MKK4 and MKK7. Active PKC*β* also acts in a feedback inhibition loop by phosphorylating Btk on serine 180. This catalyzes the removal of Btk from the plasma membrane away from its substrate, PLC*γ*2.

What follows from this proximal signaling is an integration step that connects the BCR to gene transcription in the nucleus (Figure 2). Active PLC*γ*2 hydrolyzes phosphatidylinositol 4,5-bisphosphate (PIP_2_) to diacylglycerol (DAG) and inositol trisphosphate (IP_3_). IP_3_ acts to release Ca^2+^ from intracellular stores within the endoplasmic reticulum and together with DAG activates classical protein kinase C isoforms, including PKC*β* [32]. As illustrated in Figure 2(a) active PKC*β* phosphorylates the Ras guanine exchange protein (RasGEF) RasGRP3 facilitating its activation by DAG to generate active Ras (GTP-Ras) [33, 34]. This mechanism of GTP-Ras production then combines with that of Son-of-sevenless (Sos) primed for activation by Grb2 interaction with BLNK to act as a rheostat and augment the production of active Ras by Sos [35], eventually leading to efficient activation of the cRaf-MEK-ERK pathway to transmit mitogenic signals to the nucleus. Activated PKC*β* is also important for JNK and NF*κ*B pathway activation in B cells undergoing BCR engagement (Figure 2(b)) [36]. A key target of PKC*β* within this mechanism is CARMA1 (CARD11) [37], and this protein together with MALT1 and Bcl10 form a complex, known as the CBM complex, that then attracts and activates transforming growth factor *β*-activated kinase 1 (TAK1) [38, 39]. TAK1 then catalyzes activation of the NF*κ*B pathway by phosphorylating IKK*β* which goes on to phosphorylate and dissociate I*κ*B*α* from RelA to allow its translocation to the nucleus. TAK1 also catalyzes activation of the JNK pathway by phosphorylating MKK6 [38–40]. Finally, active PKC*β* can phosphorylate Btk on S^180^, and this promotes removal of Btk from the plasma membrane by interfering with the ability of its PH domain to bind PIP_3_ (Figure 2(b)). In this way, active PKC*β* provides feedback inhibition of BCR signaling [41].

BCR engagement also results in cytoskeletal changes that facilitate formation of the immunological synapse and induce adhesion (Figure 1(d)) [42, 43]. The RacGEF function of Vav is stimulated by Syk-induced phosphorylation when it locates to the plasma membrane in conjunction with LAB, BLNK, and PIP_3_ [26, 27, 42]. The generation of active, GTP-loaded, Rac1 and Rac2 by Vav is then responsible for inducing the cytoskeletal changes that accompany BCR engagement [43]. Rac2 is essential for the activation of integrin lymphocyte functional antigen 1 (LFA1) during BCR engagement, and this is important for cell adhesion [42]. Both Rac1 and Rac2 are required to catalyze efficient F-actin reorganization required for BCR internalization [26]. Active Rac1 and Rac2 generated by BCR cross-linking may also play an important role in activating the p38 MAPK pathway through interaction with MEKK3 [44].

Protein tyrosine phosphatases (PTPs) play an important role in the BCR signaling pathway [45]. CD45 and CD148 play overlapping functions in BCR signaling and catalyze the activation of Lyn by removing phosphate from the C-terminal inhibitory tyrosine residue [46]. Such removal catalyzes a structural change in Lyn that results in autophosphorylation of the activating tyrosine residue, and this stabilizes the SFK into its active state [47]. Thus, signaling is initiated when CD45/CD148 is excluded from areas of BCR clustering (synapse formation) [48] through a process that is thought analogous to a model of kinetic segregation proposed to occur in T cells [49].

More distal to the BCR are PTPN22, SHP-1, PTPN2, and PTP-PEST, which are primarily involved in downregulation of BCR signaling. PTPN22 forms a complex with the C-terminal SFK kinase (Csk) [50], and together these proteins likely act to deactivate Lyn and Fyn where PTPN22 initially dephosphorylates the activating tyrosine residue of these SFKs and Csk then acts to phosphorylate the inhibitory tyrosine residues [51]. PTPN22 is also reported to associate and dephosphorylate E3-ubiquitin ligase c-Cbl to potentially regulate its function in controlling Syk and Lyn stability [52–54]. SHP-1 can also dephosphorylate the activating tyrosine residue of SFKs, but it has additional substrates such as ITAMs, ZAP70, Syk, and the BCR adaptor proteins BLNK and LAB [55]. Principally, SHP-1 is activated by coming into contact with phosphorylated tyrosines within immune-receptor tyrosine-based inhibition motifs (ITIMs) which are located on the cytoplasmic tails of cell surface proteins such as CD22 and CD32 [55]. Less is known about the functions of PTP-PEST and PTPN2 in B cells; PTP-PEST seems to downregulate BCR signaling by interacting with the adaptor protein Shc [56], whereas PTPN2 may have a role to play in regulating the activation of SFKs [57].

It is assumed that many of the signaling pathways initiated by BCR engagement in normal B cells are also initiated in CLL cells [6]. To a large extent this assumption can be substantiated; however, there are subtle differences that need to be taken into consideration. Importantly, BCR engagement elicits heterogeneous signaling responses with CLL cells from some cases having little or no response compared to cells from other cases [58, 59]. This heterogeneity has been linked to disease prognosis [60], and, in general, UM-CLL cells have greater capacity to respond to BCR cross-linking than do M-CLL cells [59, 61]. Moreover, the ability to respond has been linked with B cell anergy, and CLL cells that do not respond to BCR engagement are thought to be anergic [59, 61–63]. Finally, CLL cells can express proteins typically found in T cells such as ZAP70 [64–66] and Lck [67–69], which can also contribute to heterogeneity of BCR signaling [64, 67, 70, 71].

## 3. Anergy in CLL

The stochastic nature of Ig gene rearrangement that takes place during B cell development leads to the generation of self-reactive B cells, and it is estimated that the prevalence of these cells at the immature B cell stage lies between 55 and 70% of the total population [72]. Many of these self-reactive B cell clones are deleted either through a process of receptor editing that alters their specificity [73] or through the induction of apoptosis [74]. The rest escape the bone marrow and their reactivity is silenced by receptor anergy whereby self-reactive B cells become tolerant to autoantigens [75]. Importantly, many of these anergic B cells eventually die in circulation because they are denied BAFF, a TNF-related factor that potently promotes B cell survival [76, 77]. If BAFF is provided, the state of anergy can be reversed, resulting in the development of autoimmunity [78, 79].

CLL can be considered a disease of autoreactive B cells because the malignant cells from approximately half of all CLL cases secrete antibodies against autoantigens [80, 81]. In fact, mutation of nonautoreactive CLL antibody sequences from M-CLL cases to germline configuration is demonstrated to confer polyreactivity and autoreactivity on the resultant antibody [82]. This fact introduces the concept of anergy to CLL cell pathobiology owing to the possibility of constitutive engagement of the BCR on CLL cells* in vivo* [63, 83, 84]. Such engagement is observed in some, but not all, cases of CLL [15, 59, 61–63, 83, 84], with affected cells showing higher levels of intracellular calcium and activation of ERK, characteristics of anergic B cells in a mouse model [85]. Similar to normal B cells [86] the effects of BCR anergy on affected CLL cells can act globally to inhibit signaling by other receptors and are demonstrated in a recent report showing that anergic CLL cells fail to migrate toward CXCL12 because endosome recycling of Rap1 is impaired [87]. Under normal conditions B cells that are anergic to soluble antigens have short* in vivo* life spans [88]. This potentially applies to our concept of anergy in CLL because the studies describing this phenomenon have all used malignant cells isolated from blood [63, 84]. When malignant cells are separated from this environment anergy is reversed, suggesting that whilst in the blood anergic CLL cells are exposed to soluble antigen. Such chronic exposure is reported to confer a survival signal on CLL cells [63, 83, 84] and potentially releases them from requiring BAFF for long-term survival while autoantigen remains present. This is an important difference to normal B cells and possibly explains the association of CLL anergy with the indolent course of disease [63, 84].

How differences between anergic CLL and normal B cells may be manifested is potentially in the way transduction of inhibitory signals is favored by constitutive BCR engagement in these cells. Active Lyn is a driver of inhibitory signaling during BCR engagement as indicated by the development of an autoimmune phenotype in Lyn-deficient mice [89, 90]. The mechanism of inhibitory signaling pathway activation during chronic BCR engagement is thought mediated by active Lyn binding to monophosphorylated ITAMs within CD79 leading to recruitment, phosphorylation, and activation of SHIP-1 and its adaptor Dok-1 [86, 91, 92]. Importantly, Syk is not activated in anergic B cells despite active Lyn [93, 94] and this is likely because of its absolute need for dual phosphorylation of the ITAM within CD79. With this established role of active Lyn in providing inhibitory signaling in anergic B cells, it is remarkable that overexpression of constitutively active Lyn also results in the development of autoimmunity, seemingly because chronic negative signals are overridden by enhanced positive signals that eventually lead to a breakdown in tolerance within this system [95]. This is important to our understanding of BCR signaling in CLL cells because Lyn is overexpressed and constitutively active in these cells [67, 96]. Constitutive activation of Lyn in CLL cells may be the result of chronic BCR engagement, but another proposed explanation is low expression levels of the protein tyrosine phosphatase PTPRO due to epigenetic silencing [97, 98]. However constitutive activation of Lyn is maintained; higher expression levels of this SFK in CLL cells are linked with progressive disease [99]. This suggests that CLL cells and B cells expressing constitutively active Lyn are similar with respect to the dominance of positive over negative BCR signaling, a notion that is strongly supported by observations of constitutively active Syk, Btk, and PLC*γ*2 in CLL cells [100–103]. Nevertheless, the presence of negative signals seems to be important for the pathogenesis of CLL. Two studies of CD5 phosphorylation in CLL cells, one showing that active Lyn mediates the recruitment of SHP-1 to tyrosine phosphorylated CD5 to contribute to apoptosis resistance in CLL cells [104] and a second showing that constitutive phosphorylation of CD5 promotes expression of genes involved in the malignant phenotype of CLL cells, suggest that such negative signaling may be mediated by CD5 [105]. Another study showing that overexpressed PTPN22 positively regulates Akt activation in CLL cells by affecting the ability of Lyn to phosphorylate CD22 and recruit SHIP-1 [106] further substantiates a role for negative signals in CLL cell survival. Finally, a recently published abstract has provided support for Lyn-mediated negative signaling in CLL pathogenesis and reported that disease development is inhibited in the Tcl-1 transgenic mouse model of CLL, a model that closely mimics UM-CLL in humans [107, 108], when Lyn is deleted [109].

The transduction of inhibitory signals by constitutive BCR engagement is not well studied in CLL cells, probably because its role in pathogenesis of the disease is only recently recognized. Active Lyn in CLL cells could regulate additional inhibitory signaling by phosphorylating ITIMs within CD32b, CD72, CD22, and Siglec-10, all of which would then attract SHP-1 and SHIP-1 to initiate this process. This notion is supported by unpublished data from this laboratory showing that CD32b is constitutively phosphorylated on Y^292^ within its ITIM in CLL cells and that this can be inhibited by treating the cells with the pan-SFK inhibitor dasatinib. CD72 is expressed on CLL cells [110], but its phosphorylation has not been previously studied. It is a ligand for CD5 and may function in a* cis*-mediated fashion to promote the constitutive phosphorylation of this latter protein that is observed in CLL cells [104, 105]. CD22 and Siglec-10 are both members of a family of proteins known as sialic acid-binding immunoglobulin-type lectins (or sialyl adhesins) and bind to glycan residues containing *α*2,6-linked sialic acid. Whereas Siglec-10 expression on CLL cells has not yet been described, CLL cells do express CD22 at variable levels between patients [111]. How expression levels of CD22 affect CLL function is not known, but phosphorylation of this protein by Lyn is described [106] and unpublished data from this laboratory supports this observation. In terms of Siglec-10, it is likely that CLL cells also express this protein because it is expressed on CD5+ B cells [112] from which CLL cells are reported to derive [113]. Both CD22 and Siglec-10 are involved in maintaining B cell tolerance and associate with the BCR to regulate Ca^2+^ release during its engagement [114]. It is therefore tempting to speculate that high levels of CD22 and Siglec-10 on CLL cells may have a role in maintaining tolerance within anergised cells.

## 4. Can Anergy Be Overcome in CLL Cells?

It is important when considering anergy in CLL that this state is mainly associated with M-CLL cells, whereas BCR responsiveness is associated with UM-CLL cells [59, 61, 83]. This consideration is important because the antigen binding region of BCR on UM-CLL cells is polyreactive, whereas the same region of BCR on M-CLL cells is monoreactive [82]. Thus, if anergy is defined as BCR tolerance resulting from constitutive engagement with antigen, it is logical to assume that the state of anergy would be more associated with UM-CLL cells owing to the polyreactivity of their BCR. This is not the case, and one reason could be that anergy is maintained in M-CLL cells because BCR has higher affinity for antigen due to the process of affinity maturation [6, 62], whereas anergy is neither induced nor maintained in UM-CLL cells because their BCR has less affinity for antigen. Evidence that affinity maturation has taken place in M-CLL cells is provided in a study suggesting that these cells are derived from a population of CD5+CD27+ postgerminal center B cells [113]. Moreover, low levels of surface Ig expressed on M-CLL compared to UM-CLL cells are consistent with the notion that M-CLL cells are anergic because maintenance of anergy in normal B cells leads to downregulation of surface Ig expression. However, Syk, Btk, and PLC*γ*2 are all reported to be constitutively active in CLL cells [100–103], a feature which argues against anergy because Syk is not active in anergic B cells [93, 94]. Furthermore, one study analyzing the glycosylation of surface IgM on CLL cells has demonstrated that expression of immature mannosylated IgM corresponds with engagement of the BCR on both CLL and normal B cells and goes on to suggest that this also corresponds to CLL cells experiencing* in vivo* BCR stimulation [15]. Importantly, this study also demonstrates that expression of immature mannosylated IgM correlates with UM-CLL cases. An acknowledged weakness of this study is that, for technical reasons, only CLL cases with high levels of sIgM could be analyzed so it is not clear whether CLL cases with low levels of surface IgM have similar glycosylation patterns. Nevertheless, high surface IgM expression on CLL cells is associated with increased ability to respond to BCR engagement [115]. This suggests that, despite constitutive engagement of BCR, anergy is lost in UM-CLL cells so that positive signals are generated that then contribute more robustly to malignant cell survival.

In this respect, expression of microRNAs (miRs) targeting proteins important for maintaining negative BCR signaling may be one adaptation [116]. miR-155 targets SHIP-1 [117] and high expression of miR-155 is associated with UM-CLL cases [118], possibly leading to diminished ability to initiate the negative BCR signaling associated with anergy [86, 91, 92]. Other epigenetic events such as gene hypo/hypermethylation could lead to promotion or suppression of particular genes involved in BCR signaling. For example, methylation of the PTPRO gene leads to reduced expression of the phosphatase coded by this gene, and this may have the consequence of promoting the generation of active Lyn in CLL cells [97, 98]. Another protein affected by gene methylation status in CLL cells is ZAP70 [119–121] whose expression is normally restricted to T cells.

**Figure 3:**
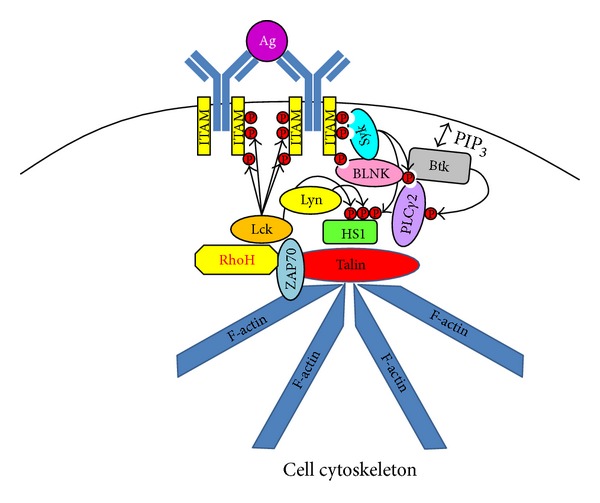
Possible roles of ZAP70, RhoH, and HS1 in BCR signaling in CLL cells. Schematic showing the potential role of ZAP70 as a scaffolding protein for Lck, RhoH, talin, and HS1 at the immunological synapse formed when the BCR is cross-linked on CLL cells. HS1 becomes hyperphosphorylated by Lyn, Syk, and Lck and plays a role with RhoH in regulating cytoskeletal remodeling.

ZAP70 expression in CLL cells was first identified in a genetic screen comparing M- and UM-CLL cells and then further characterized as a marker of poor disease prognosis [66, 122, 123]. In particular, high ZAP70 expression is observed in UM-CLL cells [65, 66, 83] where it appears to enhance BCR signaling [64, 70, 71, 124, 125] and relieve the effects of anergy on chemokine-induced migration [126, 127]. Importantly, ectopic expression of ZAP70 in M-CLL cells enhances BCR signaling strength in these cells through a mechanism that is independent of both its kinase function and c-Cbl binding capacity [70, 71]. This suggests that ZAP70 may act as an adaptor protein and restore or enhance BCR signaling in anergic CLL cells (Figure 3). It may do this by associating with talin in order to facilitate the interaction between integrin and F-actin and restore migration to chemokines such as CXCL12 [128] and possibly also to enhance BCR-mediated cell adhesion through a mechanism involving PLC*γ*2 and Btk [129, 130].

Figure 3 also illustrates that ZAP70 can associate with the GTPase RhoH [131], which plays an important role in antigen receptor signaling in T cells by recruiting both ZAP70 and Lck to the immunological synapse [132]. Expression of RhoH is a pathogenetic feature of CLL cells because deletion of this protein within the Tcl-1 transgenic mouse model of CLL delays development of the disease by affecting both BCR signaling [132] and cell migration/adhesion [133]. An additional component of this complex is hematopoietic lineage cell-specific protein 1 (HS1) whose phosphorylation and function in T cells depend on ZAP70 and Lck (Figure 3) [134]. HS1 is highly expressed in CLL cells [135–137] where it contributes to the cytoskeletal remodeling required for cell movement and migration [138]. HS1 is a substrate of Syk and Lyn in B cells [139, 140] and of Lck in T cells [134, 141] that are undergoing antigen receptor stimulation, and phosphorylation of HS1 activates its function [134, 139, 141]. High levels of phospho-HS1 in CLL cells are correlated with poor disease prognosis [136], but it is suggested in this case that hyperphosphorylation of HS1 deactivates its function because deletion of this protein within the Tcl-1 mouse model of CLL accelerates disease progression [138]. Experiments using the pan-SFK inhibitor dasatinib suggest that Lyn is responsible for constitutive phosphorylation of HS1 in CLL cells [142], which makes sense because Lyn is constitutively active [96], but other SFKs such as Lck could be additionally involved because these cells both express Lck [68, 143, 144] and use it for mediating BCR signaling [67]. Thus, considering that RhoH expression correlates with ZAP70 in CLL cells, a plausible mechanism is that it coordinates ZAP70 and Lck to the BCR to facilitate hyperphosphorylation of HS1 during BCR engagement and thereby release CLL cells from the effects of anergy and promote increased cell mobility. Once CLL cells move into circulation they would then recover from antigen engagement and upregulate surface IgM and CXCR4 expression potentially enhancing their ability to reenter tissues and undergo proliferative/prosurvival signals that include BCR engagement as has been proposed [115, 145].

Other mechanisms facilitating the generation of positive BCR signals in CLL cells may also operate. At least one study has shown that CLL cells lacking ZAP70 can still signal via BCR engagement [83]. However UM-CLL cells overcome anergy to become able to signal through the BCR; there are still elements that remain unaccounted for. One such element is that BCR engagement on CLL cells does not induce activation of the JNK pathway regardless of whether the cells respond or not [146]. This is important to our understanding of anergy in CLL because the JNK pathway is not activated in BCR-stimulated normal anergic B cells [85]. As mentioned earlier, PKC*β* is a critical mediator of JNK pathway activation in B cells through its ability to interact with and activate CARMA1 during BCR engagement [39]. The gene for PKC*β* codes for 2 splice variants, PKC*β*I and PKC*β*II [147], and work from this laboratory have shown that PKC*β*II is overexpressed in CLL cells [148]. The isoform of PKC*β* mediating CARMA1 phosphorylation in B cells has not been clearly identified [37], so it is conceivable that low expression of PKC*β*I compared to PKC*β*II in CLL cells [148] limits the ability of the former to phosphorylate CARMA1 and initiate JNK signaling.

It is also possible that anergy is masked in CLL by other factors. Thus, within the model of BCR signaling presented in Figure 1, when overexpressed PKC*β*II is activated in CLL cells, the strength of induced BCR signals is reduced due to PKC-mediated phosphorylation of Btk and downregulation of the signal. VEGF stimulation of CLL cells stimulates PKC*β*II activity, and this results in phosphorylation of Btk and inhibition of BCR signal strength [149]. Considering that CLL cells release autocrine VEGF and that high levels of this growth factor are present in lymph and splenic tissue where CLL cells proliferate [150, 151], it is entirely possible that BCR signaling is diminished when CLL cells are in tissues. When CLL cells emerge from tissues the effects of VEGF stimulation would wear off and result in apparent relief of BCR anergy.

## 5. Targeting BCR Signaling in the Therapy of CLL

**Figure 4:**
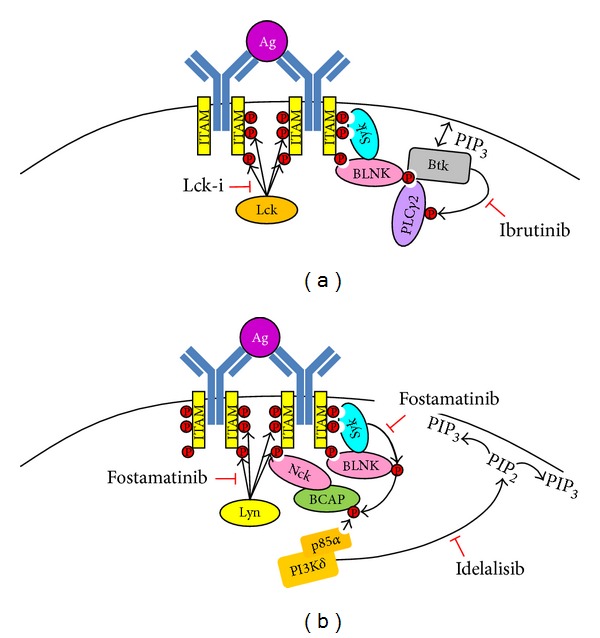
Points of inhibition for ibrutinib, idelalisib, fostamatinib, and Lck-i in the BCR signaling pathway. Illustration of proximal signals initiated during BCR engagement and points where well described inhibitors act. (a) Lck-i acts to inhibit the most proximal signaling event of SFK-mediated phosphorylation of tyrosine residues within CD79. Ibrutinib acts to inhibit the ability of Btk to phosphorylate and activate PLC*γ*2. (b) Fostamatinib acts to inhibit Syk kinase activity as well as that of Lyn. Idelalisib works to inhibit PI3K*δ* to limit the formation of PIP_3_ and has the effect of blocking membrane interaction of signaling proteins containing PH domains such as PDK1, Akt, and Btk.

Observations that Syk and Btk are constitutively active in CLL cells [100–103] have prompted the development of new agents targeting the BCR signaling pathway, and inhibitors of Btk, Syk, and PI3K*δ* have seen application in the therapy of CLL [4, 152–154]. Of particular interest is ibrutinib (Figure 4(a)), which is a new class of compound that irreversibly blocks the kinase activity of Btk by covalently modifying Cys481 [155]. The presence of ibrutinib in CLL cell cultures is reported to induce apoptosis [156] and to also inhibit the prosurvival effects of BCR cross-linking and coculture with supporting nurse-like cells [3]. This effect of ibrutinib seems attributable to its ability to inhibit Btk because siRNA knockdown of this protein in CLL cells promotes apoptosis [100]. When used* in vivo* ibrutinib exhibits a profound effect and promotes rapid resolution of lymphadenopathy coupled with lymphocytosis of CLL cells [152, 157], presumably by inhibiting the ability of BCR to activate integrin *α*4*β*1 and maintain the adhesion required for their residence within proliferation centers [3, 130]. One report has suggested that ibrutinib also blocks* in vivo* cell proliferation based on studies of CLL cells derived from patients in phase 1/2 clinical trials [158], while studies employing the Tcl-1 mouse model of CLL have shown that the presence of ibrutinib delays disease development [3, 100]. How anergy applies within the context of therapeutic response to ibrutinib is not clear, but it is suggested that a phenomenon defined as prolonged lymphocytosis, where white cell counts have not returned to normal or <50% of baseline within 12 months of treatment, is the result of ibrutinib treatment when CLL cells are anergic [157]. Nevertheless, the high clinical efficacy, coupled with relatively low toxicities [152, 159], has accelerated this compound through to regulatory approval for the treatment of mantle cell lymphoma.

Inhibition of Syk with fostamatinib (Figure 4(b)) has also shown some promise in* in vitro* studies [4, 102, 103, 160], and this has led to the development of second-generation compounds that are reported to show greater specificity [161]. A recent study of CLL cells derived from patients enrolled in a phase 1/2 clinical trial of fostamatinib has shown that this compound effectively blocks Btk activity and other BCR signaling targets in order to inhibit cell proliferation in treated patients [154], and an early report suggests that it is an effective treatment for this disease [4]. Active Syk contributes to CLL cell survival by regulating Mcl1 expression, and this was demonstrated using fostamatinib and siRNA knockdown of Syk expression [103, 162]. Fostamatinib is a competitive inhibitor of ATP and therefore limits the kinase activity of Syk [163]. However, fostamatinib is not strictly specific for Syk and other tyrosine kinases such as Lyn and Lck are also inhibited [163]. In this regard, fostamatinib is similar to the pan-SFK inhibitor dasatinib which has also been shown to have some efficacy as a single agent in the treatment of CLL [164]. This similarity extends to suggestions on their clinical use in combination with other agents; both fostamatinib and dasatinib are reported to work better when used in combination with fludarabine because of the potential influence of microenvironment in providing antiapoptotic signals [102, 165]. Importantly, inhibition of Syk blocks CLL cell migration and adhesion [160, 166], and this may explain why reduction in lymphadenopathy is observed when this agent has been used in clinical trials.

Upstream of Btk is PI3K*δ* (Figures 1(a), 1(b), and 4(b)), which is a class Ia PI3K isoform whose expression is restricted to lymphoid cells and which plays an important role in B cell development [167, 168]. In terms of CLL PI3K*δ* is an attractive target because of the nonredundant role it plays in relation to PI3K*α* and *β* with respect to BCR signaling [168]. An inhibitor for PI3K*δ* has been developed (Cal-101/GS-1101/idelalisib) and preclinical studies have shown this compound is cytotoxic and blocks BCR signaling in CLL cells [5, 169]. Like ibrutinib, idelalisib induces lymph node shrinkage and transient lymphocytosis when given* in vivo* [5]. Moreover, idelalisib might be highly suited to the treatment of CLL because it is reported to affect MZ and B-1 B cell function and reduce autoantibody response [170]. In clinical trials idelalisib shows antitumor activity when given as a single agent [171] and in combination with rituximab has been shown to significantly improve overall survival among patients with relapsed CLL [172].

Lck may also be a good therapeutic target in CLL (Figure 4(a)). It plays a major role in proximal and distal BCR signaling events in CLL cells [67], and an available inhibitor, 4-amino-5-(4-phenoxyphenyl)-7H-pyrrolo[3,2-d]pyrimidin-7-yl-cyclopentane (Lck-i), is described to be highly selective in its ability to inhibit this SFK [173–175]. Indeed, when used at concentrations that inhibit BCR signaling to ERK, Akt, and IKK in CLL cells, this compound has no effect on the kinase activity of Lyn [67]. Moreover, the presence of Lck-i in CLL cell cultures does not affect constitutive phosphorylation of Tyr^292^ and Tyr^822^ within the respective ITIMs of CD32b and CD22, nor does it affect BCR-induced tyrosine phosphorylation of CD5 [176]. This suggests that Lck operates as a differential between negative and positive BCR signaling events in CLL cells, and inhibition of its activity would therefore allow negative BCR signals to dominate. Because of its proximal role in BCR signaling in CLL cells, the effects of Lck inhibition should be similar to idelalisib and ibrutinib and reduce lymphadenopathy and promote lymphocytosis.

Finally, argument can be made for targeting PKC*β* in the treatment of CLL. Although experiments have shown that inhibition of this PKC isozyme is not overtly cytotoxic to CLL cells [148], the fact that it is potentially regulating Btk function may be important to their pathophysiology [148, 149]. PKC*β* phosphorylates Btk on S^180^ and facilitates its removal from the plasma membrane in activated B cells [41]. Inhibition of PKC*β* in CLL cells may allow for greater membrane localization of Btk and make it more susceptible to agents such as ibrutinib. Thus, as a combination therapy PKC*β* inhibitors may find use in the treatment of CLL.

## 6. Conclusions and Perspectives

It is clear that signals generated by the BCR play an important role in malignant cell survival and disease progression in CLL. It is now demonstrated that agents targeting this pathway are effective in the treatment of this disease. However, we do not yet fully understand the mechanism of BCR signaling in CLL cells, and new approaches are needed in this quest. Phosphoproteomic analysis of proteins using stable isotope labelling in culture (SILAC) may be one approach whereby a more complete picture can be made [177]. Indeed, one review has proposed that such an approach may be highly useful in the search for biomarkers in leukaemia and lymphoma [178]. One distinct advantage of this approach is the ability to decipher how agents targeting the BCR signaling pathway work. Alternatively, this approach may also give insight into the model of anergy that applies to CLL pathophysiology. Thus, with new understanding of how BCR signaling works and how it might be targeted in CLL cells, new therapies can be developed for the effective treatment of this disease.
